# Brain Region–Specific Decrease in the Activity and Expression of Protein Kinase A in the Frontal Cortex of Regressive Autism

**DOI:** 10.1371/journal.pone.0023751

**Published:** 2011-08-31

**Authors:** Lina Ji, Ved Chauhan, Michael J. Flory, Abha Chauhan

**Affiliations:** 1 NYS Institute for Basic Research in Developmental Disabilities, Staten Island, New York, United States of America; 2 The State Key Lab of Pharmaceutical Biotechnology, College of Life Sciences, Nanjing University, Nanjing, People's Republic of China; University of California San Francisco, United States of America

## Abstract

Autism is a severe neurodevelopmental disorder that is characterized by impaired language, communication, and social skills. In regressive autism, affected children first show signs of normal social and language development but eventually lose these skills and develop autistic behavior. Protein kinases are essential in G-protein-coupled, receptor-mediated signal transduction and are involved in neuronal functions, gene expression, memory, and cell differentiation. We studied the activity and expression of protein kinase A (PKA), a cyclic AMP–dependent protein kinase, in postmortem brain tissue samples from the frontal, temporal, parietal, and occipital cortices, and the cerebellum of individuals with regressive autism; autistic subjects without a clinical history of regression; and age-matched developmentally normal control subjects. The activity of PKA and the expression of PKA (C-α), a catalytic subunit of PKA, were significantly decreased in the frontal cortex of individuals with regressive autism compared to control subjects and individuals with non-regressive autism. Such changes were not observed in the cerebellum, or the cortices from the temporal, parietal, and occipital regions of the brain in subjects with regressive autism. In addition, there was no significant difference in PKA activity or expression of PKA (C-α) between non-regressive autism and control groups. These results suggest that regression in autism may be associated, in part, with decreased PKA-mediated phosphorylation of proteins and abnormalities in cellular signaling.

## Introduction

Autism spectrum disorders (ASDs) are neurodevelopmental disorders characterized by impairment in social interactions and verbal/non-verbal communication skills, and restricted, repetitive and stereotyped patterns of behavior [1]. According to a recent report from the Centers for Disease Control and Prevention, the prevalence of ASDs is 1 in 110 for children 8 years of age [2]. The symptoms of ASDs are typically present before the age of 3 years, and are often accompanied by abnormalities in cognitive functioning, learning, attention, and sensory processing. While the causes of ASDs remain elusive, ASDs are considered to be heterogeneous and multifactorial disorders that are influenced by both genetic and environmental factors. The onset of autism is gradual in many children. However, in regressive autism, children first show signs of normal social and language development but lose these developmental skills at 15–24 months and develop autistic behavior [3]. The reported incidence of regressive autism varies in different studies from 15% to 62% of cases [4]–[7]. In a few cases, regression may significantly affect language, with lesser impact in other domains such as social interaction or imaginative play [4], [8]. On the other hand, some children may regress especially in social functions and not in language [9].

Protein kinases are known to play important roles in cellular signaling pathways and are involved in brain development [10]–[13]. Protein kinase A (PKA) is a cyclic adenosine monophosphate (cAMP)–dependent protein kinase that is involved in cognitive functions and memory formation [14]–[18]. PKA consists of regulatory (R) and catalytic (C) subunits. Three genes encode for catalytic units (Cα, Cβ, and Cγ), and four other genes encode for regulatory units (RIα, RIβ, RIIα, and RIIβ) of PKA. PKA remains catalytically inactive when the levels of cAMP are low. The concentration of cAMP rises upon activation of adenylate cyclase by G protein-coupled receptors, and/or inhibition of cyclic nucleotide phosphodiesterase (PDE) enzyme. Under these conditions, cAMP binds to two binding sites on the regulatory subunits of PKA, which results in the release of the catalytic subunits. These free catalytic units of PKA can then phosphorylate proteins by transferring a phosphate group from ATP. Several studies have implicated the role of PKA in neuropsychiatric disorders such as schizophrenia, bipolar affective disorder, obsessive compulsive disorder, and panic disorders [19]–[22]. To date, no studies of PKA have been done in autism.

The intracellular levels of cAMP are controlled by PDE, which degrades the phosphodiester bond in cAMP. PDE regulates the localization, duration, and amplitude of cAMP signaling within subcellular domains. Multiple forms of PDEs have been identified on the basis of substrate specificity. PDE4, 7, and 8 act on cAMP; PDE5, 6, and 9 act on cyclic guanosine monophosphate (cGMP); whereas PDE1, 2, 3, 10, and 11 act on both cAMP and cGMP. Recent evidence has suggested altered levels of PDE4 in the brains of individuals with autism [23].

Because the levels of PDE4 are altered in autism, and PKA is involved in neuropsychiatric disorders, it was of interest to compare the activity and protein levels of PKA in different brain regions in autism (regressive and non-regressive) and age-matched control subjects. Our study suggests that PKA activity and expression are decreased in the frontal cortex of individuals with regressive autism as compared with control subjects. Such changes were not observed in individuals with non-regressive autism.

## Materials and Methods

### Autism and Control Subjects

Samples of postmortem frozen brain regions, i.e., the cerebellum, and the cortices from the frontal, temporal, parietal, and occipital lobes from autistic (N = 7–10 for different brain regions) and age-matched, typically developed, control subjects (N = 9–10) were obtained from the National Institute of Child Health and Human Development (NICHD) Brain and Tissue Bank for Developmental Disorders at the University of Maryland, Baltimore, MD. The age (mean ± S.E.) for autistic subjects was 12.6±3.2 years, and for control subjects, 12.4±3.3 years. All brain samples were stored at −70°C.

The case history and clinical characteristics for the autism and control subjects are summarized in Table 1. Donors with autism had met the diagnostic criteria of the Diagnostic and Statistical Manual-IV for autism. The Autism Diagnostic Interview-Revised (ADI-R) test was performed for the donors UMB #s 4671, 4849, 1174, 797, 1182, 4899, and 1638 (Table 2). Each donor's impairments in social interaction, qualitative abnormalities in communication, and restricted, repetitive and stereotyped patterns of behavior are consistent with the diagnosis of autism, according to the results of the ADI-R diagnostic algorithm. All donors with autism exceeded the cut-off score in these parameters. The diagnosis of autism was assigned to donor UMB # 1349 after extensive evaluation of behavioral tests, including the Autism Diagnostic Observation Schedule (ADOS), Vineland Adaptive Behavioral Scale (VABS), and Bayley Scales for Infant Development-II (BSID-II). In addition to the ADI-R, UMB # 4849 was also evaluated by the BSID-II and Childhood Autism Rating Scale (CARS), which indicated moderate to severe autism, and autism in UMB # 4671 was also verified by the VABS and BSID-II. Table 3 shows scores for the VABS test, which assesses adaptive behavior in four domains: communication, daily living skills, socialization, and motor skills.

**Table 1:** Case history and clinical characteristics of autism and control donors of brain tissue samples.

Brain tissue (UMB #)	Diagnosis	Autism Diagnostic tests	Age (y)	Sex	PMI (h)	Regressive autism	Other medical conditions	Medications	Cause of death
4671	Autism	ADIR, VABS, BSID-II	4.5	F	13	No			Multiple injuries from fall
1349	Autism	ADOS, VABS, BSID-II	5.6	M	39	Yes			Drowning
4849	Autism	ADIR, BSID-II, CARS	7.5	M	20	Yes	Lead poisoning		Drowning
1174	Autism	ADIR, VABS	7.8	F	14	No	Seizures	Depakote, Tegretol	Multiple-system organ failure
4231	Autism		8.8	M	12	No	Hyperactivity	Zyprexia, Reminyl	Drowning
797	Autism	ADIR	9.3	M	13	No	Attention deficit disorder, migraine headache	Desipramine	Drowning
1182	Autism	ADIR	10.0	F	24	Yes			Smoke inhalation
4899	Autism	ADIR	14.3	M	9	Yes	Seizures	Trileptal, Zoloft,Clonidine, Melatonin	Drowning
1638	Autism	ADIR	20.8	F	50	Yes	Seizures, Attention deficit hyperactivity disorder	Zoloft, Zyprexa, Mellaril, Depoprovera	Seizure-related
5027	Autism	WISC-R, Bender-Gestalt	38.0	M	26	No		Respirdal, Luvox	Obstruction of bowel
4670	Control		4.6	M	17				Commotio Cordis from an accident
1185	Control		4.7	M	17				Drowning
1500	Control		6.9	M	18				Motor vehicle accident
4898	Control		7.7	M	12		Hyperactive disorder	Concerta, Clonidone	Drowning
1708	Control		8.1	F	20				Motor vehicle accident
1706	Control		8.6	F	20		Congenital heart disease with heart transplant		Rejection of cardiac allograft transplantation
1407	Control		9.1	F	20		Asthma allergies	Albuterol, Zirtec, Alegra, Rodact, Flovent, Flonase	Asthma
4722	Control		14.5	M	16				Motor vehicle accident
1846	Control		20.6	F	9				Motor vehicle accident
4645	Control		39.2	M	12				Arteriosclerotic heart disease

ADI-R: Autism Diagnostic Interview Revised.

ADOS: Autism Diagnostic Observation Scale.

VABS: Vineland Adaptive Behavioral Scale.

BSID-II: Bayley Scales of Infant Development-Second Edition.

CARS: Childhood Autism Rating Scale.

WISC-R: Wechsler Intelligence Scale for Children-Revised.

**Table 2:** Autism Diagnostic Interview-Revised test scores in donors of brain tissue samples.

Autism Diagnostic Interview-Revised (ADI-R)^a^							
Diagnostic Algorithm	Cutoff score for autism	UMB 4671	UMB 4849	UMB 1174	UMB 797	UMB 4899	UMB 1638
Impairments in reciprocal social interaction (Scores:0–30)	10	26	22	22	24	22	21
Abnormalities in communication:							
Verbal (Scores:0–26)	8	-	18	-	20	-	-
Non-verbal (Scores: 0–14)	7	13	N/A	11	13	14	11
Restricted, repeated and stereotyped behavior (Scores: 0–12)	3	3	8	6	6	8	7
Abnormalities of development evident at or before 36 months	1	5	3	5	-	4	5

a: Higher score represents greater impairment.

UMB 1182: ADI-R was conducted but the scores are not available. The donor met the criteria for a diagnosis of autism.

**Table 3:** Vineland Adaptive Behavioral Scales diagnostic test for autism in donors of brain tissue samples.

Vineland Adaptive Behavioral Scales (VABS)^a^							
	UMB 1349				UMB 4671		UMB 1174
	At age: 25 months		At age: 33 months		At age: 39 months		At age: 6.4 y
Domain (Scores:20–160)	Standard Score	Age equivalent performance	Standard Score	Age equivalent performance	Standard Score	Age equivalent performance	Standard score
Communication	57	9 months	69	18 months	52	10 months	41
Daily living skills	65	16 months	62	16 months	54	14 months	22
Socialization	60	9 months	71	17 months	51	4 months	52
Motor skills	-	-	-	-	65	24 months	-
Composite	-	-	-	-	51	13 months	35

a: Higher score represents better function.

According to the medical histories for UMB-4231 and UMB-5027, the donors had psychological evaluation, and met the criteria for a diagnosis of autism. Detailed information is not available.

In this study, the subjects with autism were divided into two subgroups: regressive autism and non-regressive autism, depending on the pattern of onset of symptoms for autism. Regressive autism is a type of autism in which early development is normal, followed by a loss of previously acquired skills. Language is the most common area that regresses; this regression can be accompanied by more global regression involving loss of social skills and social interest. On the other hand, in non-regressive autism, the child never gains normal language and social skills, and initial symptoms are delayed speech development, and/or delay in development of social skills and in nonverbal communication. These children do not demonstrate regression in terms of loss of language or social skills.

#### Ethics statement

This study was approved by the Institutional Review Board (IRB) of the New York State Institute for Basic Research in Developmental Disabilities. The IRB reviewed this study in accordance with New York State Regulations and the HHS Office for Human Research Protections, including the “Human Subject Decision Chart 1,” and found that *the research does not involve human subjects* because “the research does not involve intervention or interaction with the individuals”, nor “is the information individually identifiable”. The subjects cannot be identified, directly or through identifiers linked to the system, and the consent is not required.

### Preparation of Brain Homogenates

The tissue samples were homogenized (10% w/v) in cold buffer containing 50 mM Tris-HCl (pH 7.4), 8.5% sucrose, 2 mM EDTA, 10 mM β-mercaptoethanol, and protease inhibitor cocktail (Sigma-Aldrich, St. Louis, MO) at 4°C. For extraction of protein kinases, the homogenates were mixed with an equal volume of extraction buffer containing 40 mM Tris-HCl (pH 7.4), 300 mM NaCl, 2 mM EDTA, 2 mM EGTA, 2% Triton, 5 mM sodium pyrophosphate, 2 mM β-glycerophosphate, 2 mM Na_3_VO_4_, 100 mM NaF, and 2 µg/ml leupeptin. The samples were allowed to stand on ice for 10 min, and then centrifuged at 135,000 g for 20 min at 4°C. The supernatants were collected, and the concentrations of total proteins in the supernatants were measured by the biocinchoninic acid protein assay kit (Thermo Scientific, Rockford, IL).

### Assay for PKA Activity

PKA activity was measured using the solid phase enzyme-linked immunosorbent assay (ELISA) kit from Enzo Life Sciences International, Inc. (Plymouth Meeting, PA). In this assay, the substrate of PKA was pre-coated on the wells of a microplate. The microplate wells were soaked with 50 µl of kinase assay dilution buffer for 10 min. The buffer was then carefully aspirated from each well, and the brain samples were added to the appropriate wells. The kinase reaction was initiated by adding 10 µl ATP, and was carried out for 90 min at 30°C. It was terminated by emptying the contents of each well. A phosphosubstrate–specific antibody was added to the wells except in blank, and incubated for 60 min at room temperature, followed by washing 4 times with wash buffer. The peroxidase-conjugated secondary antibody was then added except in blank, and incubation was done for 60 min at room temperature. The wells were again washed 4 times with wash buffer. The color was developed with tetramethylbenzidine substrate, and it was proportional to the phosphotransferase activity of PKA. The reaction was stopped with acid-stop solution, and the absorbance was measured at 450 nm in a microplate reader. The absorbance was divided by the concentration of total protein (µg) in each sample, and the data are represented as relative PKA activity.

### Western Blot Analysis

Total protein (15 µg) from brain homogenates of subjects with regressive- and non-regressive autism or control subjects was separated using a 10% sodium dodecyl sulfate-polyacrylamide gel electrophoresis, and then transferred to a nitrocellulose membrane. The membrane was blocked with Tris-buffered saline containing 5% fat-free dried milk for 2 h at room temperature, and further incubated overnight at 4°C with polyclonal antibody against C-subunit (isoform α) of PKA (Cell Signaling Technology Inc., Danvers, MA). The membrane was then washed 3 times with TBS-0.05% Tween 20, and incubated with horseradish peroxidase-conjugated secondary antibody for 2 h at room temperature. The membrane was washed again, and the immunoreactive protein was visualized using enhanced chemiluminescent reagent. Because PKA (C-α) and β-actin have similar molecular weights (42 KDa), polyclonal antibody against PKA (C-α) was stripped from nitrocellulose membrane, and the membrane was re-probed with monoclonal antibody against β-actin (loading control). The densities of all protein bands were measured by NIH Image J software, and the density of PKA (C-α) band was normalized with the density of β-actin for each sample.

### Statistical Analysis

Initially, autistic and control cases were collected as age-matched pairs. As data for both members of a pair were not available in all cases, and data were approximately normally distributed, unpaired two-tailed t-tests were employed to make comparisons of PKA activity in various brain regions, and of overall PKA density between autistic vs. control cases. Comparisons among controls and autistic cases showing or not showing clinical signs of regression in function were made using one-way analysis of variance (ANOVA). To guard against type I error, a Bonferroni adjustment for multiple comparisons was made to the t-tests of multiple brain regions, and for the pairwise *post-hoc* t-tests comparing each pair of the three groups that were compared in the overall ANOVA. For purposes of this adjustment, tests of different hypotheses, i.e., of activity levels and of protein contents of PKA, were not considered to be multiple comparisons.

## Results

### PKA Activity in Different Brain Regions of Individuals with Autism and Age-Matched Control Subjects: Relationship with Regression in Autism

**Figure 1:**
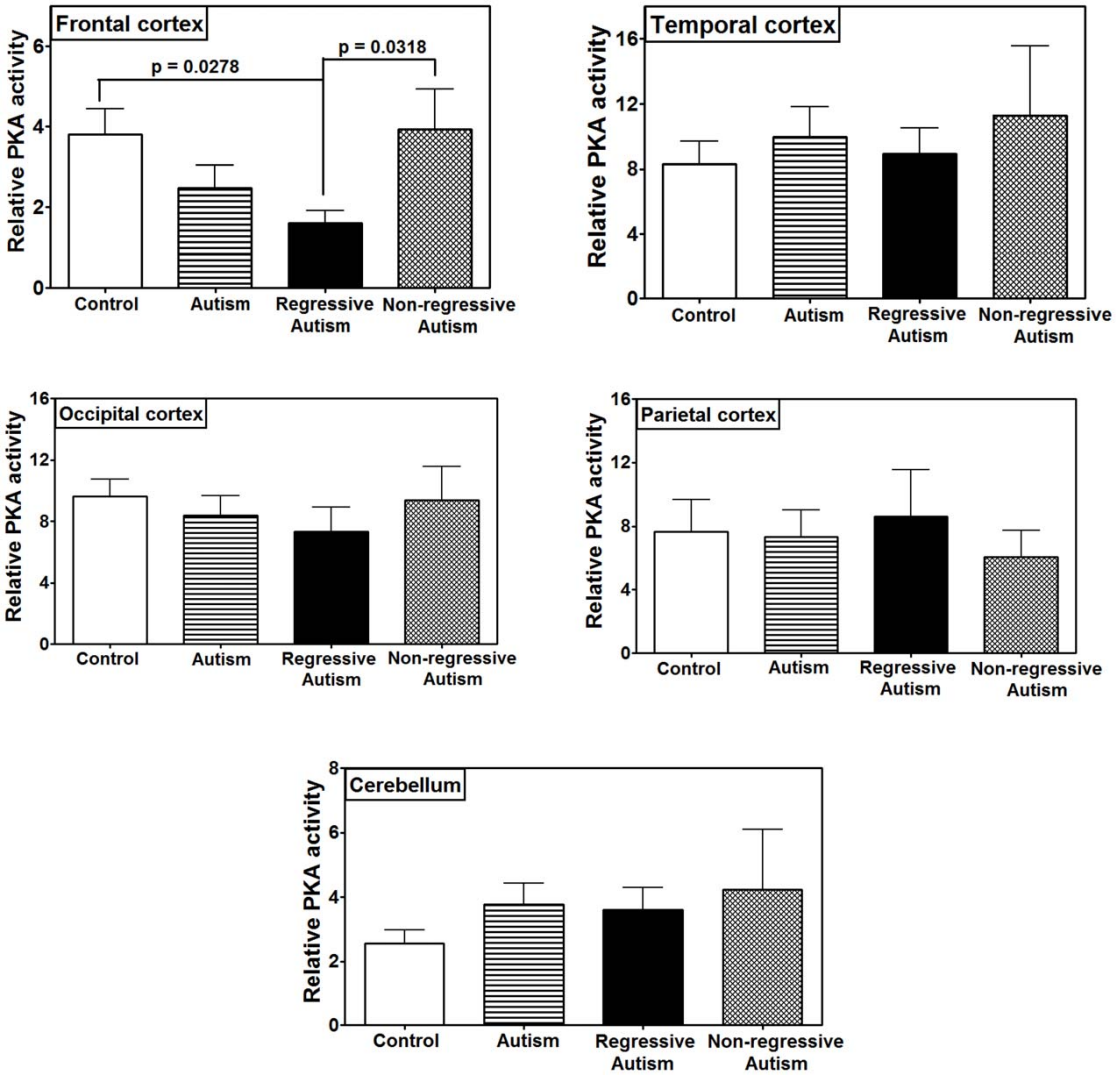
PKA activity in different brain regions from regressive autism, non-regressive autism, and age-matched control subjects. The autism group comprises combined regressive and non-regressive autism sub-groups. Brain homogenates were prepared, and activity of PKA was measured as described in Materials and Methods. Data represent mean ± S.E.

The activity of PKA was measured in the brain homogenates from the frontal, temporal, occipital, and parietal cortices, and the cerebellum in autistic and control subjects (Fig. 1). When all autism cases (regressive and non-regressive) were compared with the age-matched control group, no significant difference was found in PKA activity in any of these brain regions, although PKA activity in the frontal cortex was found to be reduced by 34.7% in the autism vs. control group. When the autism group was divided into two sub-groups (regressive and non-regressive), depending on whether there was a clinical history of regression or not, unadjusted two-tailed t-test showed a significant decrease in PKA activity in the frontal cortex of individuals with regressive autism as compared to the developmentally normal control group (p = 0.0278) and the non-regressive autism group (p = 0.0318), but these differences did not remain significant after application of the adjustment for multiple comparisons. The mean ± S.E. of PKA activity in the frontal cortex was: 2.48±0.57 in autism (regressive+non-regressive), 1.60±0.31 in regressive autism, 3.94±0.99 in non-regressive autism, and 3.80±0.65 in control groups. The alteration in PKA activity was specific to the frontal cortex in regressive autism because it was not observed in other regions of the brain, i.e., the cerebellum and the temporal, parietal, and occipital cortices, suggesting that the changes observed in PKA activity were brain region– specific in regressive autism. PKA activity was also similar in all of the brain regions between non-regressive autism and control groups.

There was no significant difference in postmortem interval (PMI) between the autistic and control groups, or between the regressive autism and non-regressive autism groups. The mean ± S.E. of PMI was: 22.0±4.2 in the autism groups (regressive+non-regressive, n = 10), 16.1±1.22 in the control group (n = 10), 28.4±7.2 in regressive autism (n = 5), and 15.6±2.6 in the non-regressive autism group (n = 5). We also studied whether there was an inverse correlation between PMI and PKA activity. Correlation analysis between PMI and PKA activity for all autistic and control subjects did not reveal any such association (data not shown). Furthermore, the cerebellum and the temporal, parietal, and occipital cortices were not affected in subjects with regressive autism in comparison with control subjects, while the frontal cortex was affected in these individuals. These results suggest that PMI was not a contributing factor to the observed alteration in PKA activity in the frontal cortex of individuals with regressive autism. There was also no significant difference in age (mean ± S.E.) between the regressive autism (11.6±2.7 years, n = 5) and non-regressive autism groups (13.7±6.1 years, n = 5).

### Protein Levels of Catalytic C-α Subunit of PKA in the Frontal Cortex of Individuals with Autism (Regressive and Non-Regressive) and Control Subjects

**Figure 2:**
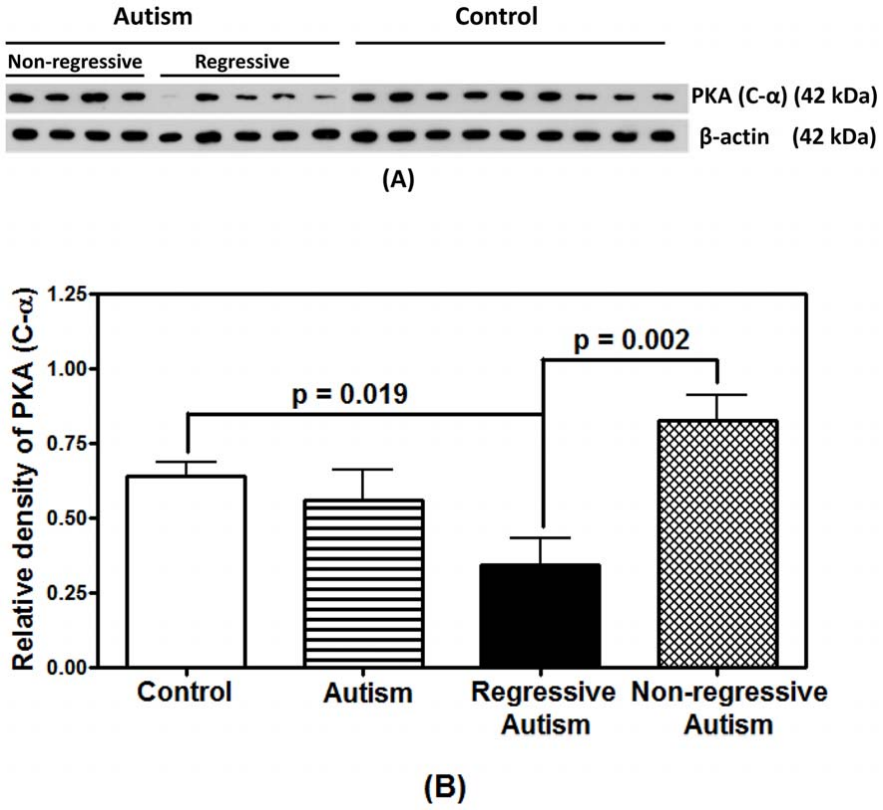
Relative protein levels of PKA (C-α) in the frontal cortex of regressive autism, non-regressive autism, and age-matched control subjects. Western blot analyses of C-α subunit of PKA in the frontal cortex of individuals with regressive and non-regressive autism, and age-matched control subjects are represented in Fig. 2A. The relative density of PKA (C-α) normalized with the density of β-actin (loading control) is shown in Fig. 2B. Data represent mean ± S.E.

Because a decrease in PKA activity was observed in the frontal cortex of subjects with regressive autism as compared to control subjects and subjects with non-regressive autism, we analyzed whether the decreased activity of PKA is related to the reduced protein contents of PKA. The protein contents of the catalytic Cα unit of PKA were analyzed in the frontal cortex of individuals with autism (regressive and non-regressive) and age-matched controls by Western blotting (Fig. 2 A). The relative densities of the protein contents of PKA (C-α) normalized with β-actin are shown in Fig. 2 B. A one-way ANOVA comparing regressive and non-regressive autism cases and controls showed a significant difference in the protein contents among these three groups (F _[df = 2,15]_ = 9.770, p = 0.002). *Post-hoc* pairwise comparisons among the groups revealed a significant decrease in the protein contents of PKA (C-α) in individuals with regressive autism (mean ± S.E = 0.34±0.09) as compared to control (mean ± S.E. = 0.64±0.05, p = 0.019, Bonferroni-adjusted) and individuals with non-regressive autism (mean ± S.E. = 0.83±0.09, p = 0.002, Bonferroni-adjusted), suggesting that the protein contents of PKA are affected in regressive autism. PKA contents were similar between non-regressive autism and control groups, and when the entire autism group (regressive and non-regressive) was compared with the control group.

## Discussion

ASDs are complex neurodevelopmental disorders. The complexity of ASDs is further increased because some affected individuals fall in the sub-group of regressive autism [7]. Behavioral changes in regressive autism fall into two broad domains: (a) loss of vocalization and (b) loss of social skills. The rate of regressive autism varies from 15% to 62% of cases in different studies [4]–[7]. While Lord *et al.* reported that 29% of the children they studied who were diagnosed with autism had lost language skills for meaningful words, and another 9% lost non-word vocalizations [5], Goldberg *et al.* reported regression in 62% of children [4]. Loss of spoken words generally associates with loss of social behavior [6], but some affected children show only loss of social skills [4]. We report here that individuals with regressive autism have decreased PKA activity in the frontal cortex of the brain. This decreased PKA activity in autistic regression may be attributed to the decreased protein contents of PKA because the protein content of PKA (C-α subunit) was also decreased in the frontal cortex of individuals with regressive autism. Interestingly, such changes were not observed in other brain regions of individuals with regressive autism, or in the frontal cortex and other brain regions of individuals with non-regressive autism. These results suggest that alterations in PKA activity and PKA expression are specific to the frontal lobe in regressive autism.

Our results suggest that PMI and age cannot account for the observed alteration in PKA in regressive autism. Other factors, such as comorbidity with seizure disorder, reported for three of 10 autism cases (of which two had regressive autism, and one had non-regressive autism), and medications, reported for two regressive autism cases, four non-regressive autism cases, and two control cases, do not seem to be contributing factors to the altered activity or expression of PKA in regressive autism. However, further studies with a larger autistic group should be done to explore this issue.

cAMP is one of the key factors for neuronal outgrowth, plasticity, and regeneration. Members of the cAMP-dependent second-messenger pathways participate in the regulation of cellular growth and differentiation and are also implicated in a variety of embryonic stages including brain development [24]. The PKA pathway is also recognized as an essential component in memory formation. Several studies in Drosophila have demonstrated the role of PKA in memory formation [25]–[29]. Mutations in the rutabaga gene, which encodes adenylate cyclase, caused significant defects in short-term memory [25]. Reduced expression or activity of DC0 (the gene encoding the catalytic subunit of PKA) caused deficits in learning, short-term memory, and middle-term memory [26]–[28]. Studies have also shown that pharmacological agents such as cAMP analogs and rolipram (an inhibitor of PDE), which are known to increase PKA activity, could improve memory [30], [31].

G-protein–coupled adenylate cyclase converts ATP to cAMP, which in turn binds to regulatory subunits of PKA. Following this event, catalytic subunits of PKA are released, which are the activated forms of PKA. PKA then phosphorylates and alters the activity of enzymes and many target proteins such as ion channels, chromosomal proteins, and transcription factors. cAMP response-binding protein (CREB) is one of the targets of PKA-mediated phosphorylation. CREB, upon activation by PKA, binds to certain DNA sequences (cAMP response elements), thereby stimulating the transcription of downstream genes and the synthesis of proteins. The CREB transcription factor is also required for long-term memory formation [32]–[34]. It is possible that a decrease in the activity of PKA in regressive autism may result in reduced phosphorylation of CREB, and thus reduced transcription and altered synthesis of some proteins.

Given that PKA is activated by cAMP, and PDE regulates the levels of cAMP, a discussion on PDE becomes imperative. Altered levels of PDE4 in the cerebella of autism subjects were reported by Fatemi and group [23]. Other studies have suggested a role of PDE4 in learning and memory in behavioral models of mice, rats, and monkeys [35], [36]. PDE4 is also reported to be involved in behavior sensitivity to antidepressant drugs in animals [37]. PDE inhibitors such as rolipram could improve object recognition [38], [39], passive avoidance [40], [41], radial arm maze [40]–[42], Morris water maze [43], and contexual fear conditioning [30], [43], [44]. PDE4 has also been studied as a potential therapeutic target for depressive disorders. It has been suggested that rolipram may have potential therapeutic benefits for major depression [45], Alzheimer's disease [36], [46], Parkinson's disease [47], [48], schizophrenia [49], [50], and tardive dyskinesia [51], [52].

Several reports suggest that some proteins related to the PKA pathway are involved in autism. Extensive evidence indicates hyperserotonemia in autism [53]–[55]. PKA regulates serotonergic activity in the brain [56]. Galter and Unsicker [57] reported that co-activation of cAMP- and tyrosine receptor kinase B (TrkB)–dependent signaling pathways plays an important role in maintaining the serotonergic neuronal phenotype. TrkB is also regulated by the cAMP/CREB pathway in neurons [58]. Furthermore, transcriptional activity of the engrailed-2 gene is also regulated by PKA [59]. The importance of engrailed can be envisioned because of its crucial roles in brain development [60] and in the development of autism [61]–[65].

In conclusion, this study suggests that the frontal cortex may be the region of the brain involved in regressive autism, where abnormalities such as decreased activity and expression of PKA can affect the signal transduction. It may have multiple effects on signal transduction pathways, which may also influence serotonergic neurons, TrkB, and engrailed-2, all of which have been suggested to be involved in the development of autism.
